# Transcriptome and Cell Physiological Analyses in Different Rice Cultivars Provide New Insights Into Adaptive and Salinity Stress Responses

**DOI:** 10.3389/fpls.2018.00204

**Published:** 2018-03-05

**Authors:** Elide Formentin, Cristina Sudiro, Giorgio Perin, Samantha Riccadonna, Elisabetta Barizza, Elena Baldoni, Enrico Lavezzo, Piergiorgio Stevanato, Gian Attilio Sacchi, Paolo Fontana, Stefano Toppo, Tomas Morosinotto, Michela Zottini, Fiorella Lo Schiavo

**Affiliations:** ^1^Department of Biology, University of Padova, Padova, Italy; ^2^Research and Innovation Centre, Edmund Mach Foundation, San Michele all’Adige, Italy; ^3^Department of Agricultural and Environmental Sciences - Production, Landscape, Agroenergy, University of Milan, Milan, Italy; ^4^Department of Molecular Medicine, University of Padova, Padova, Italy; ^5^Department of Agronomy, Food, Natural Resources, Animals and the Environment, University of Padova, Padova, Italy

**Keywords:** *Oryza sativa* (rice), salt stress, RNA sequencing, ion transporters, H_2_O_2_, salt tolerance mechanisms

## Abstract

Salinity tolerance has been extensively investigated in recent years due to its agricultural importance. Several features, such as the regulation of ionic transporters and metabolic adjustments, have been identified as salt tolerance hallmarks. Nevertheless, due to the complexity of the trait, the results achieved to date have met with limited success in improving the salt tolerance of rice plants when tested in the field, thus suggesting that a better understanding of the tolerance mechanisms is still required. In this work, differences between two varieties of rice with contrasting salt sensitivities were revealed by the imaging of photosynthetic parameters, ion content analysis and a transcriptomic approach. The transcriptomic analysis conducted on tolerant plants supported the setting up of an adaptive program consisting of sodium distribution preferentially limited to the roots and older leaves, and in the activation of regulatory mechanisms of photosynthesis in the new leaves. As a result, plants resumed grow even under prolonged saline stress. In contrast, in the sensitive variety, RNA-seq analysis revealed a misleading response, ending in senescence and cell death. The physiological response at the cellular level was investigated by measuring the intracellular profile of H_2_O_2_ in the roots, using a fluorescent probe. In the roots of tolerant plants, a quick response was observed with an increase in H_2_O_2_ production within 5 min after salt treatment. The expression analysis of some of the genes involved in perception, signal transduction and salt stress response confirmed their early induction in the roots of tolerant plants compared to sensitive ones. By inhibiting the synthesis of apoplastic H_2_O_2_, a reduction in the expression of these genes was detected. Our results indicate that quick H_2_O_2_ signaling in the roots is part of a coordinated response that leads to adaptation instead of senescence in salt-treated rice plants.

## Introduction

Soil salinity is a major constraint for crop production worldwide, particularly on agricultural land close to the sea (Zhu, 2001; Tester and Davenport, 2003). Salinization affects at least 33% of arable land, and more areas are expected to deteriorate in the coming years because of global climate changes (FAO and ITPS, 2015; FAO, 2016).

High salinity imposes osmotic and ionic stress on plants. Osmotic stress is established early after the onset of the stress, whereas ionic stress depends on ion accumulation in aerial parts of the plant and thus takes a longer time (Munns and Tester, 2008; Munns and Gilliham, 2015). The osmotic pressure at the roots level causes water loss and reduced turgor, which in turn leads to a decrease in cell expansion and plant growth. Ion toxicity, in particular due to Na^+^ accumulation, affects cellular metabolism, photosynthesis and induces oxidative stress. At the whole plant level, a reduction in the growth rate and stomatal closure are the most common outcomes in response to osmotic stress. In the case of a prolonged stress, however, these mechanisms can lead to an initial biomass loss, caused by carbon starvation, followed by early senescence events leading to cell death due to ion toxicity. Recent studies demonstrate that plants undergo anticipated senescence in response to a moderate stress, in order to convey recycled nutrients to the reproductive organs and then to guarantee the survival of next generations (Sade et al., 2017). This mechanism on the other hand, results in yield losses for annual crops.

At the cell level, Na^+^ accumulation in the cytosol causes membrane depolarization, protein misfolding and K^+^ and water loss. Successful strategies for salt tolerance probably rely on the maintenance of a high [K^+^]/[Na^+^] ratio in the cytosol, mediated by ion transporters and channels, and osmolyte production (i.e., proline) (Hasegawa et al., 2000).

Plants have evolved diverse mechanisms to cope with different aspects of salt stress. How plants sense and signal salt stress is largely unknown. However, recent evidence proves that mechano-sensitive ion channels of the hyperosmolarity-gated calcium-permeable channel (OSCA) family (Yuan et al., 2014; Zhu, 2016) may be involved in the perception of its osmotic component. In fact, specific calcium signatures have been detected in early responses to salt stress (Kurusu et al., 2015), followed by a Ca^2+^-mediated apoplastic oxidative burst (mediated by NADPH oxidases, Dubiella et al., 2013), which is likely involved both in local and long-distance signaling (Miller et al., 2010; Gilroy et al., 2014; Choi et al., 2017).

Reactive oxygen species (ROS) generated in response to an abiotic stress, besides their well-known toxic effects, have been recently recognized as also playing a role in the complex signaling network of plant stress responses, in particular in early signaling events (Julkowska and Tesreink, 2015). In order to allow ROS to be signaling molecules, non-toxic levels need to be maintained in plant tissues. This implies a tight regulation between ROS production and ROS-scavenging pathways (You and Chan, 2015).

Salinity tolerance is thus a complex trait in which different components are involved in counteracting all the changes induced by salt stress. This complexity is reflected in the lack of commercially available salt-resistant crops. Despite the high number of published works reporting the salt resistance of engineered plants under controlled conditions (Ahmadi et al., 2011; Oomen et al., 2012; Platten et al., 2013), few data are available to demonstrate the productivity and resistance of such crops in the field (Roy et al., 2014; Mickelbart et al., 2015; Hanin et al., 2016).

Further investigations into tolerance mechanisms are thus needed to identify new mechanisms of salt stress responses and to provide essential information for the production of salt-resistant crops (Ismail and Horie, 2017). Developing a rice variety with an improved level of tolerance to salinity can significantly contribute to maintaining high levels of productivity. This is especially the case considering that about 75% of the world’s rice is produced in irrigated paddy fields (Bouman et al., 2007), most of which are near river deltas, which are currently challenged by the serious threat of increased soil salinization^1^ (Jan 2018).

Exploring the genetic variability with the identification of salt tolerant/sensitive couples is possible in rice. A comparison between two or more genotypes with contrasting salt responses is beneficial in the discovery of new tolerance mechanisms in rice. Many studies have compared indica and japonica varieties, and some tolerance mechanisms have been reported, thus demonstrating the usefulness of this approach (Gregorio et al., 2002; Flowers, 2004; Ismail et al., 2007; Mohammadi-Nejad et al., 2010; Roy et al., 2014).

In this work, two varieties of rice belonging to the japonica group, which show a strong difference in tolerance to salinity, were selected and analyzed to reveal the mechanisms responsible for salt tolerance. The comparison was performed in the vegetative stage, which is one of the two growth phases most sensitive to salt in rice. Analysis of tolerance mechanisms during the most sensitive stages of growth is considered essential to provide new information on how to generate salt-resistant rice plants (Ismail and Horie, 2017).

The two varieties were initially physiologically characterized to identify the tolerance mechanisms implemented by the tolerant variety. The photosynthetic efficiency was evaluated using PAM imaging, a technique that enables the whole leaf to be examined and therefore to assess the extent of damage caused by the salt, and if necessary to see whether the damage is recoverable when the salt is removed from the culture medium. We also examined the allocation of sodium and potassium in roots and single leaves by ionomics to assess whether the tolerance was due to a recovery mechanism of ion homeostasis. We therefore performed a molecular analysis using RNA-seq to identify the metabolic and signaling pathways responsible for the physiological responses observed in the two varieties.

To reach a more complete understanding of the salt tolerance mechanism, the role of early signaling pathways induced by salt was investigated in the roots of both varieties, by analyzing the intracellular profile of the H_2_O_2_ along with the expression of genes known to be involved in the salt stress response.

## Materials and Methods

### Plant Material and Morphological Analyses

Seeds of the Italian rice varieties Baldo (B) and Vialone Nano (VN) (*O*. *sativa* L. spp. *japonica* “temperate”) ^2^ were dehulled, sterilized for 1 min in 70% ethanol and rinsed five times with deionized water. Seeds were sown on water-wetted filter paper in Petri dishes and left to germinate for 48 h at 24°C in the dark. Uniformly germinated seedlings were transferred to agar-filled (0.55% w/v, Sigma–Aldrich, Germany) seed-holders in an Araponics system ^3^ with a modified Hoagland solution (Hoagland and Arnon, 1938; Supplementary Table S1, final volume 1.9 L, static). Plants were grown until the vegetative stage V2 (collar formation on the second leaf; Counce et al., 2000) at approximately 6 days in a growth chamber at 26/21°C, with a 16/8 h photoperiod, an approximate RH of 70%, and light of 120–150 μmol photons m^-2^ s^-1^.

Seedlings at the V2 stage were grown with or without the saline solution (NaCl:MgSO_4_:CaCl_2_:NaNO_2_ = 10:2:1:1). Leaves (blade + sheath), stems and roots (thoroughly washed) were collected. For ion content analysis, samples collected at different times (1, 3, and 7 days) from control and treated plants were dried at 40°C for 48 h and stored in plastic boxes. For RNA-seq analysis and qPCRs, samples (leaves and roots) were collected at different timepoints and frozen in liquid nitrogen.

### Photosynthetic Parameters Evaluation

*In vivo* chlorophyll fluorescence measurements were performed with an imaging apparatus (FluorCam FC-800; Photon Systems Instruments, Brno, Czechia). Plants were analyzed 4 h after switching on the light and were dark adapted for 30 min before collecting photosynthetic data in order to maximize the oxidation of the photosynthetic electrons transport chain. The chlorophyll fluorescence value of dark-adapted samples (F_0_) was measured applying a non-actinic white light source (intensity < 0.05 μmol photons m^-2^ s^-1^). The maximum chlorophyll fluorescence value (F_m_) was instead measured applying a saturating light pulse (intensity = 3500 μmol photons m^-2^ s^-1^ and 800 ms duration).

PSII functionality was expressed as PSII maximum quantum yield (Φ_PSII_) and was calculated according to Maxwell and Johnson (2000). PSII quantum yield was monitored over time, using control plants not exposed to salt as the reference. Leaves were then exposed to an actinic light of 500 μmol photons m^-2^ s^-1^ for 5 min to evaluate non-photochemical quenching (NPQ) activation kinetics. Later, the light was switched off for 3 min to evaluate NPQ relaxation. NPQ parameter was also calculated according to Maxwell and Johnson (2000). All photosynthetic data in this work are presented as average ± SD of six biological replicates.

### Stomatal Aperture Measurements

The analysis was performed on the 2nd leaf of plants at stage V2, treated or untreated for 24 h as described above. About 20 stomata/leaf were imaged (Leica 5000b, Leica Microsystems, Wetzlar, Germany, 100X objective). The stomatal aperture was measured as the ratio between width and length of the stomata, as shown in Supplementary Figure S1. ImageJ2 was used for measurements (Rueden et al., 2017). Data are presented as mean ± SD of six biological replicates.

### Relative Water Content

5 cm^2^ sections of the second leaf were cut with scissors and immediately weighed (W). Then, the sections were hydrated in water in 15 mL test tubes for 4 h in a growth chamber under light. After rapid drying, samples were weighed to obtain the turgid weight (TW). Dry weight was measured after oven-drying at 40°C for 48 h.

Relative water content was determined using the following formula (Barrs and Weatherley, 1962):

(1) $$\begin{array}{c} \mathrm{RWC}(\%)\;=\;[(W-DW)/(TW-DW)]\;\times \;100 \end{array}$$

Data are presented as mean ± SD of six biological replicates.

### RNA Sequencing and Data Analyses

#### RNA Purification and Sequencing

After 3 days of treatment, roots and leaves were collected from 6 plants/experiments (*n* = 3) and homogenized in liquid nitrogen. 100 or 200 mg of sample (for leaves and roots, respectively) were used for total RNA extraction (RNeasy Plant Mini Kit followed by in-column DNase treatment, Qiagen, Hilden, Germany). 4 μg of total RNA with a RIN ≥ 8 (Bioanalyzer 2100, Agilent Technologies, Santa Clara, CA, United States) were sent to the IGA Technology Services ^4^ (Udine, Italy) for library preparation (TruSeq Stranded mRNA, Illumina) and sequencing on HiSeq 2000 platform (single-read 50bp, 6-plex, about 20 million reads/sample) (Supplementary Figure S2). The datasets generated for this study can be found in the Gene Expression Omnibus (GEO ^5^) under accession number GSE109341.

#### RNA-seq preprocessing

According to Finotello et al. (2014), Illumina raw reads were preprocessed with FASTX Toolkit 0.0.13.2.^6^ The overall quality of preprocessed results was then manually inspected using the quality reports generated with FastQC.^7^

Preprocessed reads were mapped with TopHat (Kim et al., 2013) on the *Oryza sativa* v. Nipponbare genome, downloaded from the MSU Rice Genome Annotation Project (version 7.0) (Kawahara et al., 2013). Gene coordinates file help also to map the reads spanning splice junctions (TopHat option ‘-G’). Reads multimapped were removed from the final results, together with those reads sharing less than 96% identity with the reference. Finally, read counts were computed using bedtools (Quinlan and Hall, 2010; Supplementary Table S2).

#### Annotation

To expand the functional annotation of rice genes, the entire set of transcripts was annotated with the Argot web server (Falda et al., 2012, 2016; Lavezzo et al., 2016), which assigned Gene Ontology terms to each input sequence (Gene Ontology Annotation database downloaded on 2013-12-29, PFAM release 27.0). This procedure provided novel annotations for many genes with unknown function (Supplementary Table S3).

#### Identification of Differentially Expressed Genes

The raw counts were used as input of a state-of-the-art differential expression analysis workflow (Anders et al., 2013), based on the R language and the edgeR (v. 3.8.6) bioconductor package (Robinson et al., 2010). Briefly, we compared the treated samples with the corresponding controls for both leaves and roots separately for the two varieties. For each comparison, only reads with at least 1 count per million (cpm) in 3 samples were included in the following Generalized Linear Model (GLM) based-pipeline. The normalization factor and the estimated dispersion were computed, both “trended” (or, whenever not possible, “common”) and “tagwise,” before fitting a GLM to each feature. Finally, we computed a likelihood ratio test and considered differentially expressed those genes having a p-value greater than 0.05 after a Benjamini and Hochberg correction for multiple testing (Benjamini and Hochberg, 1995).

#### Pathway Enrichment Analysis

Rice genes were assigned to clusters of orthologous groups from the KEGG database using blastKOALA (Kanehisa et al., 2016; Supplementary Table S4). This information together with the data matrix of the count-per-million (cpm) value of all the genes was the input for the Pathway Enrichment step, which was performed using Pathway Inspector (Bianco et al., 2017). Briefly, the pathway information was downloaded from the KEGG database (Release 78.1, May 1, 2016) and parsed reconstructing gene networks. According to Sales et al. (2012), genes were directly connected when there was an intermediate interacting element (e.g., a chemical compound not measured), and complexes were expanded in groups of interacting nodes (cliques). This information on pathway topology was used as input in the Differential Expression Analysis of Pathways (DEAP) (Haynes et al., 2013). The DEAP algorithm was initially developed for microarray experiments; thus, we fed it with normalized cpm after log2-transformation (performed using the voom bioconductor package (Law et al., 2014)). Through this approach, the expression data were combined with the topology: the differential expression was computed for all possible paths within the graph. The type of relationship (catalytic or inhibitory) determined the summand sign. Each pathway was then assigned with the maximum absolute value of the differential expression among all its paths, which was then used to test the entire pathway using a random rotation approach (Langsrud, 2005). We performed 100 rotations and identified the enriched pathway as those obtaining p-value smaller than 0.05 in the rotation test. For each enriched pathway, also the corresponding most differentially expressed path was identified.

#### RNA Sequencing Data Validation

We tested the expression pattern of 20 DEGs from the RNA profiling experiment using an OpenArray-based nanofluidic RealTime-PCR technique (see qPCR Section of Materials and Methods). A comparison of the data from the two approaches is shown in Supplementary Table S5.

### Determination of Ion Contents in Leaves and Roots

Leaves (blade + sheath), stems and roots (washed carefully) were collected after 1, 3, and 7 days of treatment, dried at 40°C for 48 h and stored in plastic boxes. Then, dry leaf and root samples were weighed and digested by a microwave digester system (MULTIWAVE-ECO, Anton Paar GmbH, Graz, Austria) in Teflon tubes filled with 10 mL of 65% HNO_3_ by applying a one-step temperature ramp (to 210°C in 10 min and maintained for 10 min). After 20 min of cooling time, the mineralized samples were transferred into polypropylene test tubes.

Samples were diluted 1:40 with MILLI-Q water, and the different elements concentration was measured by inductively coupled plasma mass spectrometry (ICP-MS; Aurora-M90 ICP-MS, Bruker Daltonics Inc., Billerica, MA, United States). A 2 mg L^-1^ aliquot of an internal standard solution (^72^Ge, ^89^Y, ^159^Tb) was added to both samples and calibration curve to give a final concentration of 20 μg L^-1^.

Typical polyatomic analysis interferences were removed using CRI (Collision-Reaction-Interface) with an H_2_ flow of 93 mL min^-1^ through a skimmer cone. Data are shown as mg of the ion per g of sample (dry weight).

### qPCR

qPCRs were performed using the QuantStudio 12K Flex real-time PCR system, both for the OpenArray technology (Thermo Fisher Scientific, San Diego, CA, United States) and the standard protocol.

TaqMan^®^ OpenArray^®^ Real-Time PCR Plate with Custom Gene Expression Assays (56 probes) was designed and purchased from Thermo Fisher Scientific. The following cycle was used: 10 min pre-incubation at 95°C, followed by 40 cycles of 15 s at 95°C and 1 min at 60°C (Stevanato et al., 2018). The list of Gene Expression Assays based on TaqMan chemistry used in this work is reported in Supplementary Table S6. Data were normalized against the average transcript abundance of 2 housekeeping genes (elongation factor 1-alpha (REFA1), Os03640561_s1, Os03g0177400; ubiquitin-40S ribosomal protein S27a-1 (UBQ), AIS09F9, Os01g0328400).

GoTaq^®^ qPCR master mix (Promega, United States) and standard qPCR protocol were used for *SERF1* (NCBI: Os05g0420300; primer for GAGTGAGGAGCTCATTGTTTACGA and primer rev ACATCAAAATTTCCATGTCATCTA); ubiquitin (NCBI: Os05g0160200) was used as reference gene (primer for TTCTACAAGGTGGACGACGC and primer rev AGATCAGAGCAAAGCGAGCA).

The comparative C_T_ method was used to analyze the gene relative expression (ΔΔC_T_ method, Livak and Schmittgen, 2001). All data are the means of three biological replicates, each one composed of nine technical replicates ± SD.

### H_2_O_2_ Imaging and Inhibition Experiments

Four-day old seedlings were treated with 100 mM NaCl in hydroponic solution. For NADPH oxidase inhibition experiments, seedlings were pre-treated for 1h with 5 μM diphenyleneiodonium (DPI, Sigma–Aldrich, Germany). Thus, roots were incubated with 10 μM DHR123 (Sigma–Aldrich, Germany) for 15 min followed by 5 min rinsing in water. Roots were imaged by using a Leica B5000 fluorescence microscope (Leica Microsystems, Germany, 2.5x objective) with an I3 filter. ImageJ2 (Rueden et al., 2017) was used for image analyses. Data are presented as the mean ± SD of 10 biological replicates. Experiments were performed three times.

### Statistical Analyses

Student’s *t*-tests were applied to experiments with a sample number greater than 30. Wilcoxon–Mann–Whitney tests were applied for *n* < 30. For the analysis of RNA sequencing data, refer to the text above.

## Results

### Salt Stress Responses in Salt Sensitive and Tolerant Rice Varieties

Among the 17 Italian varieties tested (Bertazzini and Forlani, 2011), we selected the two that showed the greatest difference in response to salt exposure: the tolerant variety, Baldo (B) and the sensitive variety, Vialone Nano (VN). Both varieties are of great economic importance for local (VN) and export (B) markets.

Rice is grown in the deltas and coastal areas in Asia and Europe. Rising sea levels due to climate change have been threatening rice cultivation in Asia^8^. In Italy, during prolonged drought, sea water creeps inland from the mouth of the Po river for many kilometers. The fresh water becomes brackish and the salt enters the ground by capillarity. In our experiments we simulated the seawater flooding of paddy fields, as often happens in the river deltas. Seawater contains mainly sodium, chloride, magnesium, sulfate and calcium, with Na^+^ and Cl^-^ being the most predominant. The saline solution used in this work was prepared by combining different salts with the following proportion: NaCl:MgSO_4_:CaCl_2_:Na_2_SO_4_ = 10:2:1:1. Seawater consists of about 35 g/l in dissolved salts, corresponding to about 500 mM NaCl. A soil becomes salty when the salt concentration reaches about 40 mM NaCl and rice sensitivity shows up at about 60 mM NaCl (Munns and Tester, 2008).

Experiments at different NaCl amounts suggested 100 mM is the best concentration to highlight differences between the two varieties without killing them in a few days. Baldo is not resistant but more tolerant, as we were unable to find resistant varieties among all the previously tested varieties.

**FIGURE 1:**
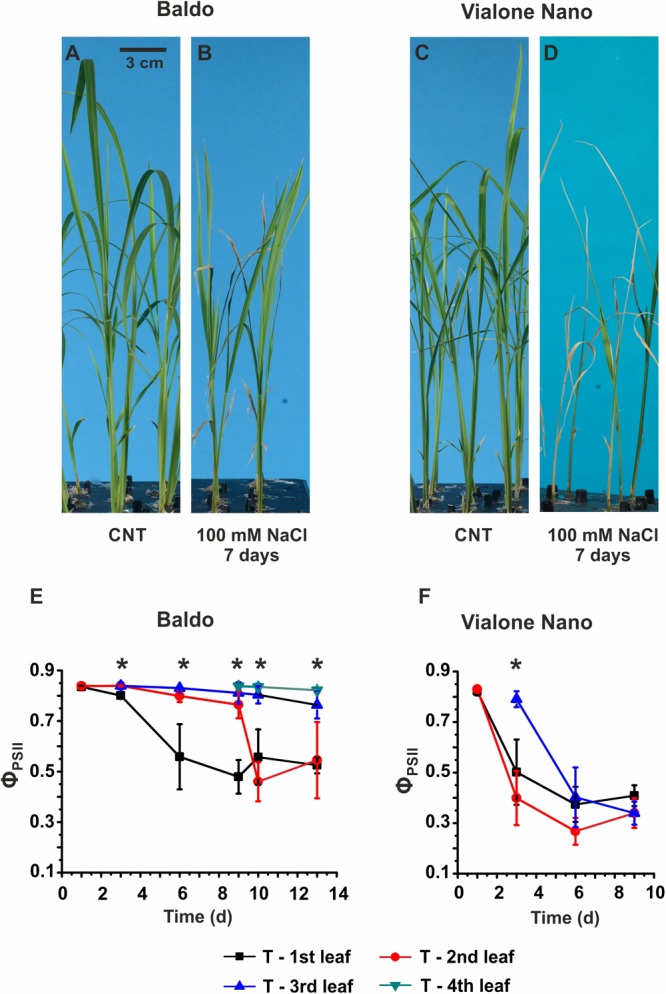
Morpho-physiological analyses of Baldo and Vialone Nano rice plants in control and stress conditions. **(A–D)** Seedlings were cultured in hydroponics up to the 2nd leaf stage and then switched to salt solution for 6 days. **(A,C)** Untreated plants. **(B,D)** Plants treated with saline solution containing 100 mM NaCl. **(E,F)** PAM imaging results for Baldo **(E)** and VN **(F)** plants, treated (T) with salt. Data are expressed as an average of six biological replicates ± SD; Asterisks indicate statistical significance for all the visible differences (*p*-value < 0.05).

The two varieties were grown in hydroponics with/without saline solution. We first characterized their physiological response (**Figures 1A–D** and Supplementary Figure S3).

In the sensitive variety, wilting of the second and third leaves was observed after 3 days of treatment (Supplementary Figure S3H), and multiple chlorotic leaves were detected after 6 days in the saline solution (**Figure 1D**). The PAM imaging technique confirmed a large decrease in PSII maximum quantum yield (Φ_PSII_) in all leaves of the sensitive plants after the 6th day (**Figure 1F**). In contrast, in tolerant plants, only the 1st and 2nd leaves were affected by salt, whereas the 3rd and the 4th leaves (**Figure 1E**), which emerged in the presence of salt, showed the same photosynthetic efficiency as the control samples (Supplementary Figure S4).

**FIGURE 2:**
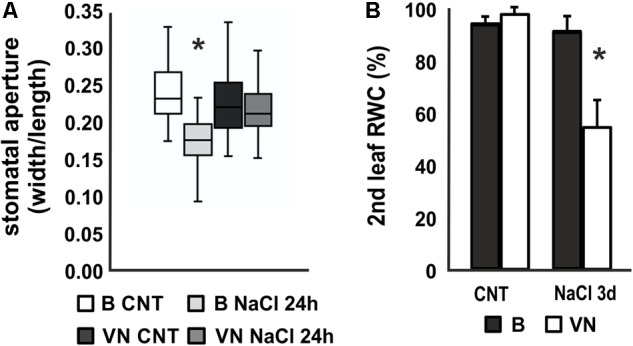
Stomatal aperture and relative water content (RWC) in plants treated with saline solution. **(A)** Box and whisker plot showing the stomatal aperture measured in the 2nd leaf of plants treated for 24 h (*n* = 20, 6 biological replicates). **(B)** Relative water content (RWC) of the 2nd leaf after 3 days of treatment (*n* = 6). Values are mean ± SD. (^∗^*p*-value < 0.01). B, Baldo; VN, Vialone Nano.

Stomatal aperture measurements after 24 h of treatment showed a reduction in the second leaf of the B plants, whereas no significant differences were observed between treated and untreated plants in VN plants (**Figure 2A**). In line with this, the relative water content (RWC) of the second leaf decreased to 54.1% in the sensitive variety after 3 days of treatment (**Figure 2B**), while in the tolerant plants no significant water loss was recorded.

**FIGURE 3:**
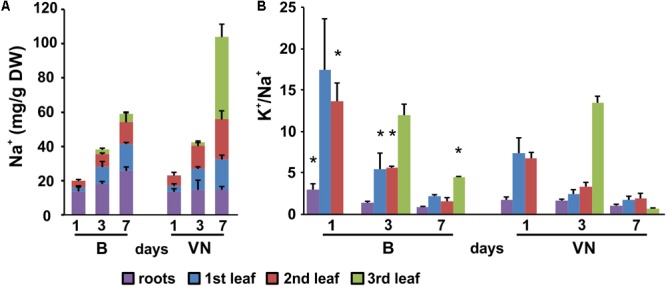
Sodium pattern distribution **(A)** and [K^+^]/[Na^+^] ratios **(B)** in roots and leaves (first, second, and third) at 1, 3, and 7 days of treatment. Data are expressed as an average of six biological replicates ± SD. B, Baldo; VN, Vialone Nano. Asterisks indicate statistical significance between Baldo and Vialone Nano, calculated for each timepoint and for each sample (root, 1st, 2nd, 3rd leaf) (*p*-value < 0.05).

The evaluation of K^+^ and Na^+^ contents by ICP-MS technique showed that in the tolerant variety, Na^+^ was restricted to the roots and older leaves, with the exclusion of salt from the third leaf (**Figure 3A**). Conversely, in the sensitive variety Na^+^ was more uniformly partitioned between the roots and shoot, and salt accumulation was observed in all the leaves (**Figure 3A**). The [K^+^]/[Na^+^] ratio was lower in the leaves of the sensitive variety than the tolerant variety (**Figure 3B**), resulting in a larger ionic stress in the susceptible plants.

**FIGURE 4:**
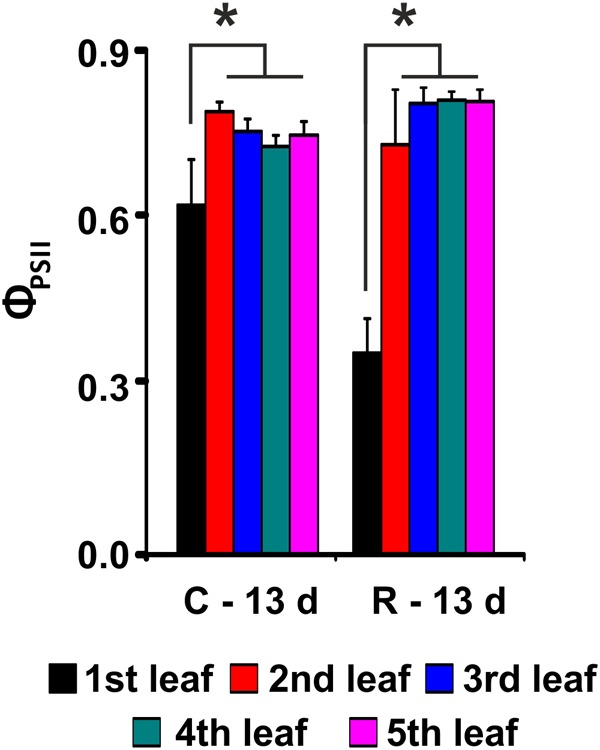
PSII maximum quantum yield (Φ_PSII_) evaluation after recovery from salt stress for Baldo plants. C – plants not exposed to salt treatment; R – plants in recovery medium. Data are expressed as an average of 6 biological replicates ± SD. Asterisks indicate statistical significance for all the visible differences (*p*-value < 0.05).

To further characterize the salt response of plants, a growth recovery experiment was conducted after 6 days of stress. After 7 days of culture in salt-free medium, the recovery capacity of the two varieties was assessed. The lengths of the 3rd and 4th leaves and the Φ_PSII_ were monitored for both varieties to evaluate the recovery of both the growth capacity and the photosynthetic performance (**Figure 4** and **Table 1**).

**Table 1:** Recovery capability of Baldo plants.

	3rd leaf length (6 + 7 days)		4th leaf length (6 + 7 days)	
100 mM NaCl	–	+	–	+
Baldo	24.2 ± 0.4	16.7 ± 0.3	28.3 ± 0.8	24.6 ± 2.1
Vialone Nano	17.9 ± .0.9	3.7 ± 2.1^∗^	20.5 ± 1.4	5.0 ± 2.8^∗^

*^∗^76% of dead plants. Length of 3rd and 4th leaves after 6 days of salt stress followed by 7 days in salt-free medium. The + column indicates seedlings under salt stress, whereas the – column indicates the corresponding controls. Values are the mean ± SD of 18 biological replicates in three technical replicates.*

Our results showed that the tolerant variety recovered completely after 6 days of treatment (**Figure 4** and **Table 1**), whereas in the sensitive variety, the percentage of dead plants reached 76% (**Table 1**), and there was a negative impact on photosynthesis in the remaining plants (not shown).

### Transcriptomic Analysis Showed Adaptation in Tolerant Plants and a Mis-Targeted Response in the Susceptible Variety

To better understand the different outcomes of the two varieties, i.e., adaptation (growth recovery) in B plants, and senescence in VN plants, transcriptomic analyses were conducted in roots and leaves 3 days after salt stress.

#### Pathway Enrichment Analysis Demonstrated That Salt Sensitivity Caused a Wide and Mistargeted Response

A total of 542,309,740 single-end, 50 nt long reads were obtained from the sequencing experiments (mean per sample of 22,596,239 with a standard deviation of 3,817,253), which decreased to 536,477,150 after pre-processing (mean per sample of 22,353,215 with a standard deviation of 3,792,380). Most of the reads (93.1%) were successfully mapped to the rice genome and met the requirements described in the Section “Materials and Methods.” Of these, 3.48% were mapped in multiple positions and were discarded.

**FIGURE 5:**
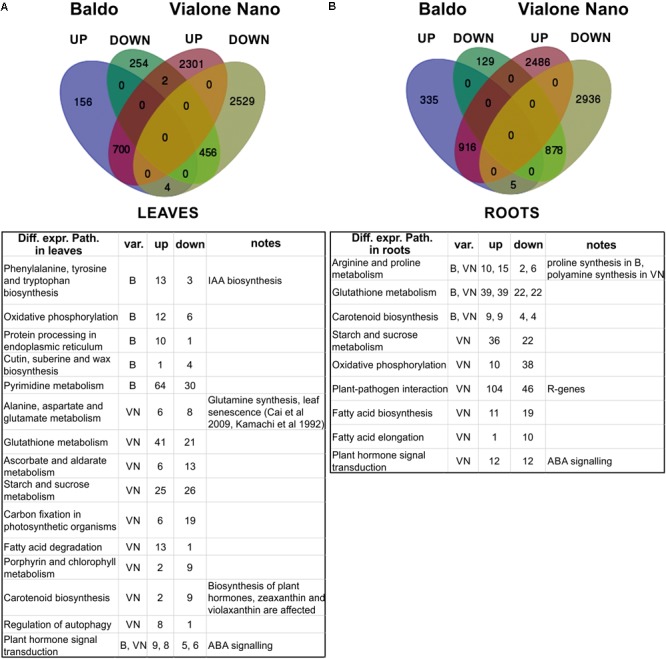
Transcriptomic analysis of leaves and roots after 3 days of salt stress. **(A,B)** Venn diagrams of up- (UP) and down (DOWN)-regulated genes (upper part). Tables (lower part) report the main differentially expressed pathways resulting from pathway enrichment analysis reported in Supplementary Tables S10, S11. B, Baldo; VN, Vialone Nano; var., variety.

Sequencing data were analyzed as described in the Section “Materials and Methods” and exploiting metabolic pathways from the KEGG database. The results are summarized in **Figure 5** and are available online at http://pathwayinspector.dmm.unipd.it/projectview/0ggWH7/ (Jan 2018).

As shown in **Figure 5** and **Table 2**, in the sensitive variety VN, a large number of genes (DEGs) was regulated both in the leaves (5992 and 1572, respectively) and roots (7221 and 2263, respectively), almost fourfold the number of B.

**Table 2:** Number of DEGs and DE pathways for each comparison.

Comparison	Diff. Expr. Genes	Diff. Expr. Pathways	Genes in Pathways
VRT vs. VRC	7221	57	1219
BRT vs. BRC	2263	23	384
VLT vs. VLC	5992	67	996
BLT vs. BLC	1572	29	435

*The following acronyms were used: V, Vialone Nano; B, Baldo; R, root; L, leaves; T, treated (3 days salt-stressed seedlings); C, control (without salt stress). The first column summarizes the results of the edgeR pipeline, whereas the last two columns summarize the enrichment analysis (performed with DEAP).*

To better interpret the many DEGs, a pathway enrichment was performed: genes were assigned to clusters of orthologous groups from the KEGG database (4th column in **Table 2**), gene networks (topology) were created, and finally the expression data were combined with the pathway topology to compute an enrichment score (see Materials and Methods for further details). We thus obtained a list of enriched pathways and their modulated components for each pairwise comparison. The number of enriched pathways was proportional to the number of DEGs and was therefore higher in the sensitive variety (**Table 2**), suggesting a wider response to salt stress in the VN variety.

**Figure 5** shows the major enriched pathways with the number of genes involved. The complete list of enriched pathways is reported in Supplementary Tables S7, S8 for leaves and roots, respectively. Some pathways (22 in leaves and 20 in roots) were common between the two varieties. Of these, a few included different DEGs (i.e., “selenocompound metabolism,” “fructose and mannose metabolism,” “pantothenate and CoA biosynthesis,” and “purine metabolism” in leaves; “arginine and proline metabolism” and “arginine biosynthesis” in roots). The redundancy of genes could account for this discrepancy because no difference was observed in the regulation of those pathways. Only two exceptions were noted: “selenocompound metabolism” in leaves, which in VN shifted clearly toward Se-Met synthesis (Supplementary Figure S5), and in “arginine and proline metabolism” in roots in which the up-regulation of proline biosynthesis in B and the up-regulation of polyamines biosynthesis in VN were observed (Supplementary Figure S6).

Regarding the pathways identified only for the B variety (two in roots and six in leaves), for “phenylalanine, tyrosine and tryptophan biosynthesis,” the up-regulation of indole (IAA precursor) synthesis was noted (Supplementary Figure S7). For “pyrimidine metabolism,” the salvage pathway appeared to be activated (Supplementary Figure S8), which has been previously observed in salt-stressed mangrove trees (Suzuki-Yamamoto et al., 2006).

Concerning only the VN variety (31 in roots, and 45 in leaves), the pathways involved in chlorophyll metabolism, carbon fixation, oxidative stress response, autophagy, and basic metabolism (glycolysis, riboflavin and protein synthesis) were affected by salt stress. In particular, in leaves, the genes involved in the photosystem assembly were down-regulated, whereas those implicated in nitrogen reallocation (i.e., glutamine synthetase) and autophagy were up-regulated, implying that the leaves were undergoing an active chloroplast dismantling followed by reassimilation of nitrogen, typical of both natural and stress-induced leaf senescence (Sade et al., 2017) (**Figure 5** and Supplementary Tables S7, S8). Of note, the “plant–pathogen interaction” pathway was enriched in VN roots (**Figure 2**), which consists of a group of 150 genes that belong mostly to the group of R genes (Supplementary Table S9), representing an example of a mis-targeted stress response in the sensitive variety.

It is now recognized that abiotic stresses lead to a reorganization of organ development in plants. Hormones are putatively involved in this response. In our system, in both B and VN varieties we found many hormone biosynthetic and signaling pathways between DEPs (carotenoid biosynthesis for ABA, diterpenoid biosynthesis for GAs, cytokinins and brassinosteroid biosynthesis) both in roots and leaves (Supplementary Tables S7, S8).

#### Analysis of Stress Responsive Genes Showed That Tolerant Plants Activate Effective Stress Responsive Mechanisms

**FIGURE 6:**
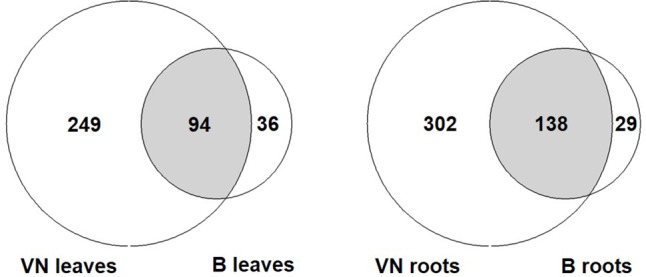
The number of DEGs related to abiotic stress responses listed in Supplementary Tables S7, S8. Few DEGs overlap between the two varieties, with VN showing a broad response, 3 to 4-fold more than in B plants. B, Baldo; VN, Vialone Nano.

In order to compare the response of the two varieties, all DEGs represented by the following GO terms were gathered: response to salt stress, osmotic stress, water stimulus, desiccation, water deprivation, and oxidative stress (**Figure 6** and Supplementary Tables S10, S11). In the sensitive variety, approximately 3- to 4-fold the number of genes found in tolerant plants were differentially expressed both in leaves and in roots (343 and 440 in VN and 130 and 167 in B, respectively). Of these, only 94 and 138 were shared between the two varieties in leaves and roots, respectively (**Figure 6**).

Among the DEGs shared between the two varieties, we found genes encoding for salt/drought-induced proteins, including aquaporins, dehydrins, late embryogenesis abundant proteins, and annexins, indicating that both varieties responded to salt. Genes involved in ROS detoxification (i.e., peroxidases and catalases) were shared only in roots, where both varieties accumulated sodium (**Figure 3A**). In leaves, these genes were differentially expressed only in the sensitive variety. These results suggested that VN plants experienced strong oxidative stress (genes for NADPH oxidoreductase, catalases, superoxide dismutases, and ascorbate peroxidases) and cellular damage (genes for DNA helicases and heat shock proteins) in both the roots and leaves (Supplementary Tables S10, S11), while B plants limited the oxidative stress to the roots. The group of DEGs that is only present in B plants comprised two genes (LOC_Os11g03440; LOC_Os12g03150), coding for putative MYB60 transcription factors, which are known to be involved in stomatal aperture in Arabidopsis and grape (Cominelli et al., 2005; Galbiati et al., 2011). The strong downregulation of these genes in B leaves might be related to stomatal closure. In addition, LOC_Os12g12580, encoding for a putative alkenal reductase, involved in defense against oxidative damage in several plant species (Mano et al., 2005), was found to be the most down-regulated gene in B plant roots.

#### Analyses of Membrane Proteins Involved in Ion Homeostasis Highlighted Susceptible Plants Suffering from Long-Term Osmotic Stress and Ion Toxicity

As reported above, the two varieties differed in terms of sodium allocation throughout the plant (**Figure 3A**). We thus gathered all the DEGs annotated as ion transporters/channels and carriers, in order to have a more detailed picture of the plants’ physiological conditions.

Abbreviations and full names of these genes, according to the Aramemnon database^9^, are reported in **Table 3**.

**Table 3:** Number of DEG encoding channels, H^+^ pumps and carriers gathered from the RNA sequencing data.

	*B*		VN			*B*		VN	
Categories	Leaf	Root	Leaf	Root	Categories	Leaf	Root	Leaf	Root
**Channels:**	**21**	**29**	**35**	**56**	**Carriers:**	**61**	**87**	**151**	**193**
OSCA-type channels	1	2	4	3	ClC family anion:proton antiporter	1	0	3	3
Ammonium transporters	2	3	1	6	SWEET-type sugar efflux transporter	4	5	8	8
SLAC-type guard cell anion channels	3	1	3	1	HKT-type potassium/sodium cation transporter	0	0	2	1
GLR-type ligand-gated cation channels	0	3	1	9	HAK/KUP/KT-type potassium cation transporter	4	6	11	14
CNGC-type cyclic nucleotide-gated cation channels	1	1	1	3	Auxin transporter	1	3	7	8
Potassium cation channels	2	3	2	4	ProT-type proline transporter	1	0	3	2
Aquaporin/small solute channels	3	4	9	14	CCC-type cation:chloride co-transporter	0	0	1	1
					monosaccharide transporters	2	8	10	20
**Active transport:**	**5**	**9**	**22**	**22**	NHX-type proton:sodium cation antiporter	0	0	2	1
V-type ATPase	0	0	9	8	CHX-type proton:monovalent cation antiporter	1	4	2	5
P-type ATPase	1	7	8	12	CAX-type proton:calcium cation exchanger	1	0	4	1
Putative proton-translocating PP-ase	2	0	3	0	CCX-type cation:calcium cation exchanger	1	0	1	2

*In accordance with the Aramemnon database (http://aramemnon.uni-koeln.de/), for each gene the full name is written immediately after the abbreviation. B, Baldo; VN, Vialone Nano.*

After 3 days of salt stress, sensitive plants were still regulating the genes encoding mostly for aquaporins, NSCCs (non-selective cation channels, e.g., ionotropic glutamate receptor GLR and cyclic-nucleotide gated channels CNGC), ATPases, high affinity K^+^ transporters (HAK), and proline and monosaccharide transporters, primarily in roots (**Table 3**). In addition, genes involved in Na^+^ and Cl^-^ transport (i.e., Na^+^/H^+^ antiporter NHX, chloride channel ClC, cation chloride co-transporter CCC, and high affinity K^+^/Na^+^ transporter HKT) were modulated only in the VN plants (**Table 3**), suggesting that susceptible plants were still suffering from osmotic stress, water deficit and ion toxicity after 3 days of salt treatment.

### A Particular NPQ Activation in the Leaves of Tolerant Plants Suggested the Involvement of the Zeaxanthin Biosynthetic Pathway

**FIGURE 7:**
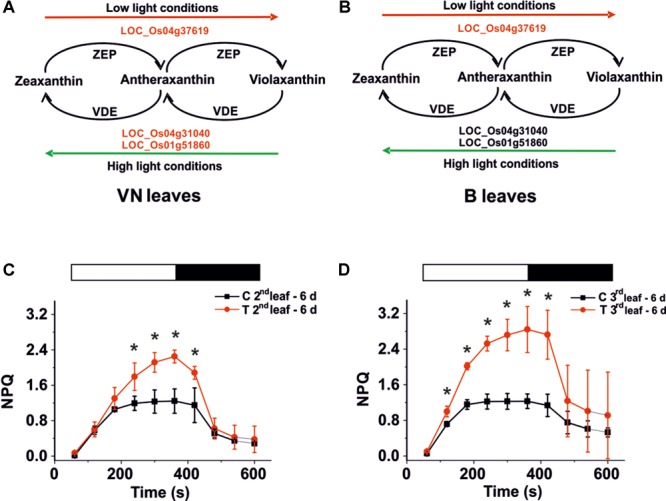
Xanthophyll cycle and NPQ analysis. Genes involved in this cycle are down-regulated (red script) in VN **(A)** but not in B **(B)** plants. **(C,D)** NPQ activation kinetics for Baldo plants exposed to salt stress. NPQ kinetics for the second **(C)** and third **(D)** leaf after 6 days of salt treatment. White bars – light induction, black bars – dark relaxation. In red, salt treated plants – T; in black, control plants – C. Data are expressed as an average of six biological replicates ± SD; Asterisks indicate statistical significance for all the visible differences (*p*-value < 0.05).

In leaves of sensitive plants, we found that genes encoding for Zeaxanthin epoxidase (ZEP) and Violaxanthin de-epoxidase (VDE) (**Figure 7A**) were down-regulated. Whereas in tolerant plants, only the ZEP encoding gene was down-regulated (**Figure 7B**). This may correlate with PAM imaging analyses that show a different ability to activate non-photochemical quenching (NPQ). In fact the regulatory mechanism of photosynthesis reduces the photo-oxidative damage caused by the over-excitation of PSII (Erickson et al., 2015), requiring the presence of Zeaxanthin. After 6 days of salt treatment, NPQ was higher in the B treated plants than in the controls (**Figures 7C,D**, red curves). Using the imaging system, we observed that the high NPQ was particularly evident in areas of the leaf that maintained their PSII functionality (Supplementary Figure S9), thus suggesting that its increase correlates with the activation of a response to salt.

By contrast, in VN plants, we did not observe any enhanced NPQ activation compared with the control plants (Supplementary Figure S9).

### A Prompt Response to Salt Stress Induces Survival Signaling Pathways

Physiological and molecular data reported to date highlight a different fate for the two varieties, adaptation versus senescence, and suggest differences in the early events induced by salt stress to explain the induction of different pathways.

#### A Fast H_2_O_2_ Burst Followed by the Expression of *SERF1* Is Observed in Roots of Tolerant Plants

Schmidt et al. (2013) described a salt-specific transcription factor belonging to the ERF (ethylene responsive factor) family, SERF1, as being early induced in rice roots upon salt stress (10 min), and demonstrated its specificity to salt. Since it is known that *SERF1* is activated by H_2_O_2_ (Schmidt et al., 2013) and rice roots generally produce H_2_O_2_ very early after salt stress (Hong et al., 2009), we investigated the role of H_2_O_2_ and SERF1 in the early phases of salt stress responses in our experimental conditions.

**FIGURE 8:**
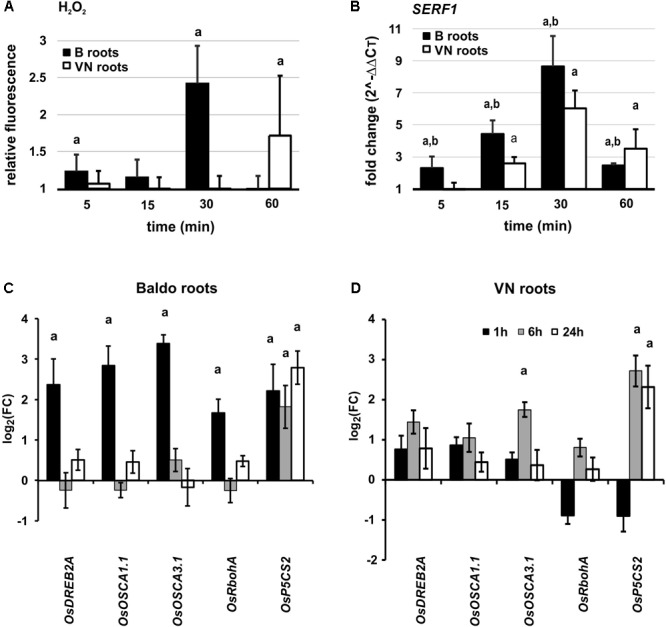
Early responses in plant roots. **(A)** Overview of H_2_O_2_ production in roots of plants exposed to salt stress. Values are calculated with respect to the control at each time-point. Data are mean ± SD of 10 biological replicates and three technical replicates. **(B)** *SERF1* expression profile at different time-points in roots. **(C,D)** Expression profile of genes involved in perception, signaling and response to salt stress in Baldo **(C)** and Vialone Nano **(D)** roots. Data are expressed as mean ± SD of three biological replicates and nine technical replicates. Letters represent *p*-value < 0.01 with respect to the control (a) or to the other variety (b).

The DHR123 probe was used to evaluate the intensity of H_2_O_2_ production at different time-points, in the roots of tolerant and sensitive plants (Supplementary Figure S10). Interestingly, we observed an increase in H_2_O_2_ already at 5 min in the tolerant variety, followed by a peak at 30 min (**Figure 8A**). In the susceptible variety, we only noted a slight increase after 1 h of treatment (**Figure 8A**).

*SERF1* was induced already after 5 min in roots of the B variety, with a peak around 30 min. In VN plants, however, the gene was up-regulated later and to a lesser extent (**Figure 8B**).

In the signaling pathways mediated by SERF1, we studied the expression profile of *OsDREB2A* (dehydration-responsive element-binding protein 2A), a gene encoding a transcription factor involved in abiotic stress response (Agarwal et al., 2006; Mallikarjuna et al., 2011), which is directly regulated by SERF1 (Schmidt et al., 2013) during salt stress. In B plant roots, *OsDREB2A* was up-regulated already at 60 min (**Figure 8C**).

Other genes, known to be involved in calcium and H_2_O_2_ signaling, showed a similar pattern, and were up-regulated earlier in the tolerant plants (**Figures 8C,D**). These genes were: *RbohA* (respiratory burst oxidase homolog protein A, Ma et al., 2012; Wang et al., 2016), involved in the signal transduction pathway; *OsOSCA1.1* and *3.1* (Li et al., 2015), involved in osmosensing; *P5CS2* (delta-1-pyrroline-5-carboxylate synthase 2, Hur et al., 2004), responsible for proline synthesis in osmotic stress responses.

#### Genes Involved in Salt-Specific Signaling and Ion Homeostasis and Compartmentalization Show an Early Induction in Roots of Tolerant Plants

As the fast recovery of a high [K^+^]_cyt_/[Na^+^]_cyt_ ratio is required as a survival strategy (Hasegawa et al., 2000), we investigated the early expression profile of genes involved in ion homeostasis.

A high [K^+^]_cyt_/[Na^+^]_cyt_ ratio can be obtained through the activity of K^+^ channels/transporters or Na^+^/H^+^ antiporters located both at the plasma and tonoplast membranes.

**FIGURE 9:**
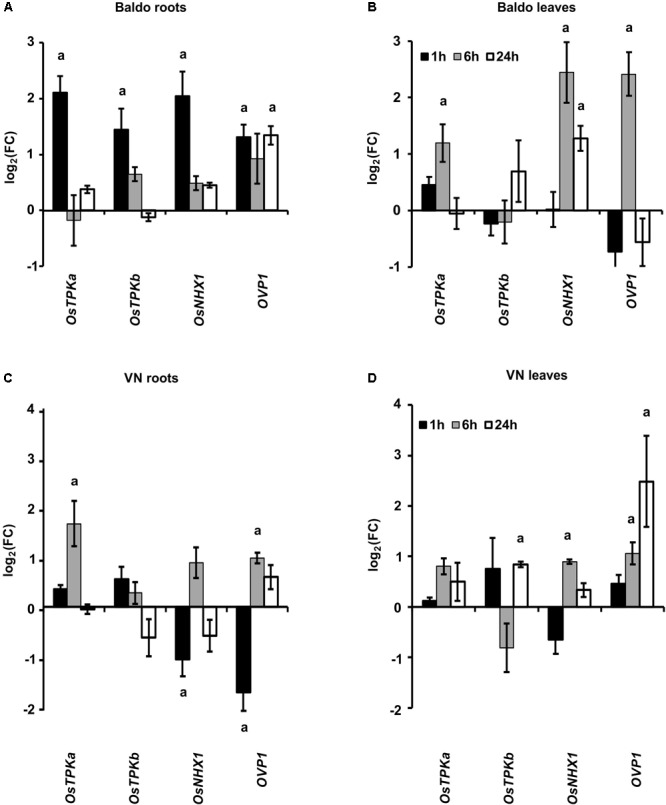
Expression profile of genes involved in K^+^ release from the vacuole (*TPKa* and *b*) and in Na^+^ compartmentalization (*NHX1, OVP1*). **(A,B)** Expression profiles in B plant roots **(A)** and leaves **(B)**. **(C,D)** Expression profiles in Vialone Nano plant roots **(C)** and leaves **(D)**. Data are expressed as mean ± SD of three biological replicates and nine technical replicates. (a, *p*-value < 0.01 with respect to the control).

As shown in **Figures 9A,B**, the gene encoding for the vacuolar cation/proton antiporter NHX1 (Fukuda et al., 2011; Amin et al., 2016) was strongly up-regulated only in B roots (1 h) and leaves (6 h) at the early stage. The expression pattern of the vacuolar H^+^-translocating pyrophosphatase OVP1 encoding gene, probably involved in the generation of the proton motive force that drives transporters activity in salt stress conditions (Gaxiola et al., 2001; Zhang et al., 2011) was also analyzed. This gene was up-regulated in the tolerant variety in roots and leaves after 1 h and 6 h, respectively (**Figures 9A,B**), whereas in VN leaves, it was up-regulated later, only 24 h after treatment (**Figure 9D**).

TPKa and TPKb, two members of the tonoplast two-pore outward potassium channel family (Isayenkov et al., 2011; Ahmad et al., 2016), were up-regulated in the first hour in the roots of B plants but only after 6 h in VN roots (**Figures 9A,C**). In leaves, no significant differences were observed between the two varieties.

The results reported in **Figure 9** clearly show that B plants had early-activated mechanisms aimed at Na^+^ compartmentalization into vacuoles, thus improving tissue tolerance to ionic stress.

#### H_2_O_2_ Plays a Role in Inducing the Early Expression of Genes Involved in Signaling and Ion Homeostasis

In order to test our hypothesis on the role of the early H_2_O_2_ burst in inducing an adaptive response in B plants, we performed experiments in the presence of the NADPH oxidase (NOX) inhibitor DPI. The inhibition of the plasma membrane NOX abolished the H_2_O_2_ peak usually observed after 30 min of treatment (**Figure 8A** and Supplementary Figure S11) and reduced the expression level of *SERF1* by 50.1%. After 1 h of treatment, the expression level of *OsDREB2A* and *NHX1*, two genes upregulated at this timepoint in the tolerant variety (**Figure 8C**), declined by 31 and 49.1%, respectively.

These results suggest that the loss of the early H_2_O_2_ peak in the tolerant variety affects the signaling pathway mediated by SERF1, which becomes more like that observed in VN roots (**Figures 8, 9**).

## Discussion

Today high salinity is one of the main constraint for crop production worldwide. The concentration of salts is continuously increasing in soils that are close to coastal areas or in areas frequently subject to drought (Zhu, 2001; Tester and Davenport, 2003; FAO, 2016). Three quarters of rice paddy fields are located in the main river deltas. Soils in these regions are rich in dissolved salts, mainly NaCl, due to either flooding or irrigation with brackish water. Rice is the most sensitive cereal to salt: the yield is affected as soon as the electrical conductivity in the soil reaches 6 dS/m (Munns and Tester, 2008; Hanin et al., 2016).

As plants are sessile organisms, they have evolved mechanisms to cope with sudden salt stress. Salinity resilience relies on different traits that were explored in the last two decades in many works (for a review see Roy et al., 2014; Hanin et al., 2016). Despite the vast amount of literature demonstrating the salt resistance of engineered plants in the laboratory, there are no significant and exploitable results on plant resilience in the field. This may be due to the persistent gap in understanding the whole picture of salt stress responses in crops. The results presented in this work, add new insights into the importance of a prompt response, mediated by H_2_O_2_, in the activation of salt tolerance mechanisms that lead to adaptation, and provide new perspectives in terms of the genetic improvement of salt tolerance.

The most explored salt tolerance trait is the ability to maintain ion homeostasis, in terms of a high K^+^/Na^+^ ratio in the cytosol and low Na^+^ content in leaves, by activating K^+^ and Na^+^ transport systems, both at the local level (i.e., root cells directly affected by the salt) or systemically (reduction of sodium in the xylematic flow, Cotsaftis et al., 2012; Roy et al., 2014; Formentin, 2017; Ismail and Horie, 2017; Kobayashi et al., 2017).

The tolerant variety Baldo (B) allocates sodium differentially throughout the plant body: more in the roots than the leaves, and more in the first leaf than the second or third. Differences in sodium allocation might explain how and why the B variety is more tolerant to ionic stress than the sensitive variety. Roots are more salt tolerant than leaves (Munns and Tester, 2008): maintaining salt in the roots as long as possible, the tolerant variety has adopted a successful strategy. As a result, in B plants, PSII functionality was affected in old leaves (i.e., the first leaf) and not in the new leaves (i.e., the third leaf). By contrast, Vialone Nano (VN) plants were strongly affected by salt treatment and showed large impairments in photosynthetic efficiency in all leaves. In addition, using an imaging system, compared with the more common pulse-amplitude modulation (PAM) technology (Zhu et al., 2011; Zulfugarov et al., 2014), we observed that photosynthetic efficiency did not decrease homogeneously in the B plants, and although some parts of the leaves were sacrificed by the plant, photosynthesis was unaffected in other parts even within the same leaf.

The sensitive variety VN did not show this response, and all parts of all the leaves were affected. These patterns were matched by the activation of specific mechanisms of sodium allocation among and within leaves, revealing that B plants likely evolved mechanisms to compartmentalize Na^+^ in contrast to VN plants. This strategy is advantageous for photosynthesis: it is better to completely sacrifice a part of a leaf and keep the rest functional, rather than lose efficiency in all cells. The recovery experiments also demonstrated the capacity of B plants to limit the damage to the first leaf, which was sacrificed in order to support the photosynthetic activity of the others and to continue the photosynthetic growth of the entire plant with the emergence of new leaves.

These results concerning the activation of the NPQ mechanism, are in contrast with previous results showing an NPQ decrease in salt-treated Arabidopsis (Stepien and Johnson, 2009) and rice plants (Zhu et al., 2011). To date, only in *Physcomitrella patens*, a representative of the early land colonization of plants, has NPQ been found to be enhanced in response to salt treatment (Azzabi et al., 2012). These results suggest that salt stress induces a general response that affects mechanisms involved in protection from oxidative stress by lowering the ROS production caused by PSII damage. The PAM technology could therefore provide an initial evaluation of salt exposure and could be performed directly in the field. An increase in NPQ activation could thus be used in combination with ΦPSII as a biomarker of the response to salt stress, providing evidence of an appropriate response in plants.

The ability of tolerant plants to retain sodium in the roots, which is referred to as tissue tolerance (Munns and Tester, 2008), is likely related to efficient sodium sequestration in root cell vacuoles (through NHX1/OVP1 coupled activity) and potassium release to the cytosol (mediated by TPKs channels). In our system, the early activation of genes encoding tonoplast transporters involved in Na^+^ sequestration into the vacuoles (NHX1 and OVP1; Gaxiola et al., 2001; Zhang et al., 2011; Barragán et al., 2012; Amin et al., 2016) and K^+^ release to the cytosol (TPKs, Maathuis, 2011) provided a strong indication that a tissue tolerance mechanism was activated in the B plants. The induction of *TPKb* only in B roots is in line with findings regarding its involvement in K^+^ homeostasis in stress conditions (Ahmad et al., 2016). Notably, NHX1, OVP1 and TPKa encoding genes were upregulated in the leaves of tolerant plants, after 6 h of stress, when we can assume that the sodium content is low. This result indicates that in tolerant plants, a rapid root-to-shoot signal is generated and triggers the pre-activation of tolerance mechanisms before the onset of ion stress in leaves.

Another trait of salt tolerance is the maintenance of osmotic pressure inside the cells. Besides the role played by ion transporters, the biosynthesis of compatible solutes (e.g., proline for rice) and stomata closure are the main mechanisms involved in limiting the loss of water (Hasegawa et al., 2000). Our results suggest that an efficient osmotic response is taking place in B plants since we observed (i) the early upregulation of the P5CS2 gene, both in roots and leaves, (ii) the downregulation of MYB60 related genes, (iii) stomatal closure, and (iv) a limited water loss in B plants.

Applying transcriptomics, we evaluated the response to stress at the molecular level after 3 days of salt exposure, when both varieties were initiating their specific programs in response to salt stress: adaptation (recovery in growth) in B plants versus senescence in VN plants. Based on the quality and quantity of differentially expressed genes (fourfold higher in VN plants), the response of the sensitive variety was broad and not specific compared to the tolerant variety. The onset of a genetically controlled cellular dismantling process (namely leaf senescence) was visible, as expected, in the VN leaves and was due to the downregulation of genes involved in carbon fixation and chloroplast assembling, and the upregulation of genes involved in nitrogen remobilization (Kamachi et al., 1992; Cai et al., 2009). In the roots, instead, we found a group of 150 DEGs involved in a plant-pathogen interaction, which can be interpreted as a misleading response of VN plants. Although abiotic and biotic stress responses share some components, e.g., signal molecules and hormones, this group of genes is composed of R-genes that are related to biotic stress.

This finding raises a question regarding the apparently altered pathway from the perception to the transduction of the stress signal in susceptible plants.

In order to answer the question, we started with the evidence that calcium and H_2_O_2_ are universal signal molecules, which transduce a stimulus after sensing. Understanding how the cell decides which pathway to activate is the next big challenge (Gilroy et al., 2014). Regarding salt stress, calcium and H_2_O_2_ waves, through OSCA hyperosmolarity-gated Ca^2+^ channels and plasma membrane NADPH oxidases (NOXs), are likely responsible for salt perception and rapid root-to-shoot signaling (Mittler et al., 2011; Gilroy et al., 2014; Evans et al., 2016; Choi et al., 2017), leading to sodium exclusion from the shoot (Jiang et al., 2012). A wrong H_2_O_2_ wave can thus affect signal transduction not only at the local level but also systemically, leading to a misleading response.

An H_2_O_2_-mediated salt-specific response has been described in rice. A new transcription factor, belonging to the class of ethylene responsive genes, named Salt-Responsive-ERF1 transcription factor (SERF1), was shown to be involved in ROS-dependent transcriptional regulation in rice roots and in root-to-shoot signaling (Schmidt et al., 2013, 2014) in response to salt.

Our findings suggest that the delayed H_2_O_2_ burst observed in roots of the sensitive variety, hampered the activation of genes involved in salt stress signaling (*SERF1, DREB2A*), ion homeostasis (*NHX1, TPKb*) and turgor maintenance (*P5CS2*), thus preventing the organization of a specific response, both in the roots and leaves. This evidence supports the hypothesis that the early H_2_O_2_ burst in roots of the tolerant variety plays a role in building a rapid and specific salt stress response.

Experiments with the NOX inhibitor DPI demonstrated that the intracellular H_2_O_2_ burst is generated at the plasma membrane level. The prevention of the intracellular H_2_O_2_ burst in B roots significantly affected the expression of downstream genes, e.g., *SERF1, DREB2A* and *NHX1*, resulting in a similar response to that observed in the VN variety.

How tolerant plants can sense and react earlier than sensitive plants is an interesting issue. As plasma membranes are involved in mediating calcium and H_2_O_2_ signals, it is possible that the two varieties have a different membrane composition. Calcium channels and NADPH oxidases are made of subunits, and it would be interesting to explore the contribution of each subunit to the signal transduction, or to investigate whether there are subunits that are more prone to respond to the stress. In this work we show that in B plants, the expression of OSCA and NOX genes (namely *OSCA1*.3, *OSCA3*.1, *RbohA*) occurred early after salt addition. The expression of these genes is not the cause but the consequence of the rapidly induced H_2_O_2_ peak in tolerant cells. However, we cannot rule out that B plants have a pre-set membrane composition that activates the adaptive mechanism well before the stress causes damage.

## Conclusion

This report points out that early H_2_O_2_ -dependent signals are part of the coordinated activation of downstream genes that trigger a specific salt tolerance response. In fact, tolerant plants rapidly perceived salt stress and responded by activating effective tolerance mechanisms, within the first 24 h. This response led to ionic stress tolerance in roots and adaptation in leaves, given by (i) the regulation of cytosolic [K^+^]/[Na^+^], (ii) differential sodium allocation throughout the plant, (iii) maintenance of PSII efficiency, (iv) NPQ increase, and (v) the appearance of new unaffected leaves.

Further studies are planned to identify all the players involved in the perception and transduction of the salt stress stimulus, for example by using genetically encoded probes for calcium and H_2_O_2_. The manipulation of early signaling events are of paramount importance for the future of plant engineering because early signals are the hub used by cells to select the right response or to cope with different stresses at the same time (Gilroy et al., 2014). Controlling this hub might improve the resilience of crops in the field, where plants are simultaneously challenged by different stresses.

## Author Contributions

EF designed the experiments and wrote the manuscript. CS and EbB performed the morphological measurements. GP and TM carried out PAM imaging analyses. SR and PF performed the pathway enrichment analyses. EL and ST handled the RNA sequencing data. EnB and GS performed the ion content measurements. PS handled the openarray data. MZ reviewed the manuscript and FLS planned the experiments, wrote and edited the manuscript.

## Conflict of Interest Statement

The authors declare that the research was conducted in the absence of any commercial or financial relationships that could be construed as a potential conflict of interest.

## Footnotes

1 http://irri.org/news/hot-topics/rice-and-climate-change

2 www.enterisi.it

3 www.araponics.com

4 www.igatechnology.com

5 https://www.ncbi.nlm.nih.gov/geo/

6 http://hannonlab.cshl.edu/fastx_toolkit/index.html

7 http://www.bioinformatics.babraham.ac.uk/projects/fastqc/

8 http://irri.org/news/hot-topics/rice-and-climate-change

9 http://aramemnon.uni-koeln.de/
