# Supplementary material for: Transcriptome and Cell Physiological Analyses in Different Rice Cultivars Provide New Insights Into Adaptive and Salinity Stress Responses
Source: Front Plant Sci. 2018 Mar 5;9:204. doi: 10.3389/fpls.2018.00204 (PMC5844958; doi:10.3389/fpls.2018.00204)
Supplement: Supplementary file 12 [file Presentation_1.pdf]

Supplementary Material

Transcriptome and cell physiological analyses in different rice cultivars provide new insights into adaptive and salinity stress responses

Elide Formentin[1]*, Cristina Sudiro[1], Giorgio Perin[1], Samantha Riccadonna[2], Elisabetta Barizza[1], Elena Baldoni[3], Enrico Lavezzo[4], Piergiorgio Stevanato[5], Gian Attilio Sacchi[3], Paolo Fontana[2], Stefano Toppo[4], Tomas Morosinotto[1], Michela Zottini[1], and Fiorella Lo Schiavo[1]

*** Correspondence:** Corresponding Author: elide.formentin@unipd.it

1 Supplementary Figures and Tables

1.1 Supplementary Figures

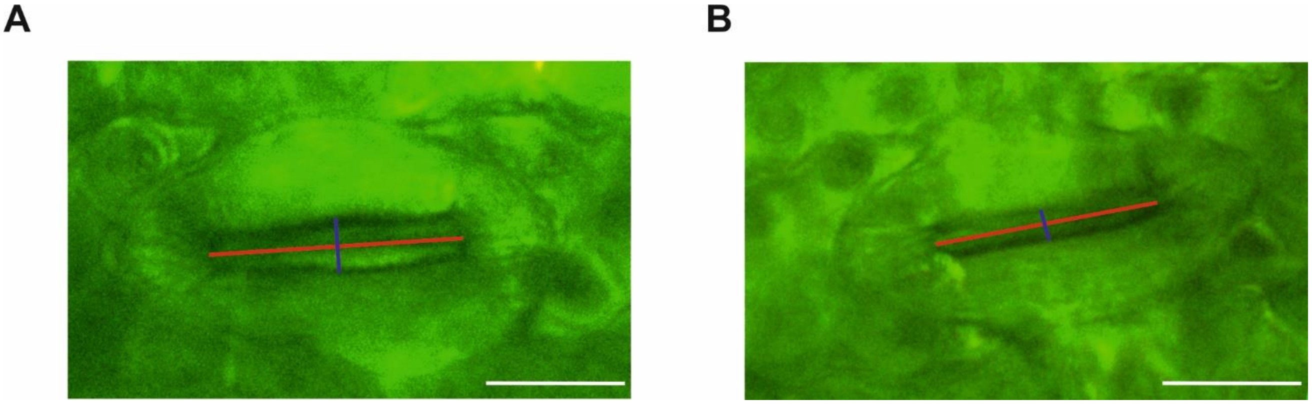

Supplementary Figure 1. Measurement of the stomatal aperture: A) example of an open stomata; B) example of a closed stomata. Width (blue line) and length (red line) were measured and stomatal aperture was calculated as the ratio of width over length. Bar = 1 μM.

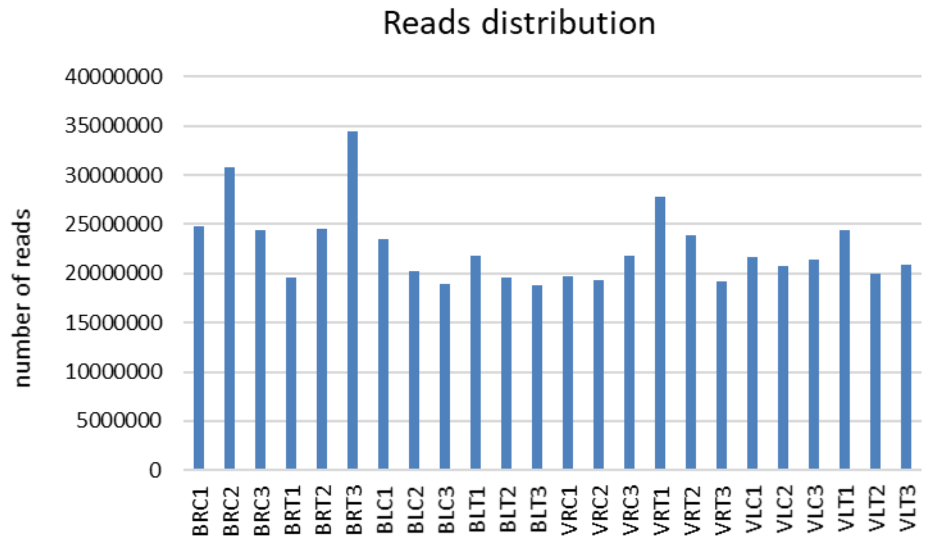

Supplementary Figure 2. Distribution of reads among samples. The following acronyms are used: V: Vialone Nano; B: Baldo; R: root; L: leaves; T: treated (3 days salt-stressed seedlings); C: control (without salt stress); 1,2,3 suffixes stand for technical replicates.

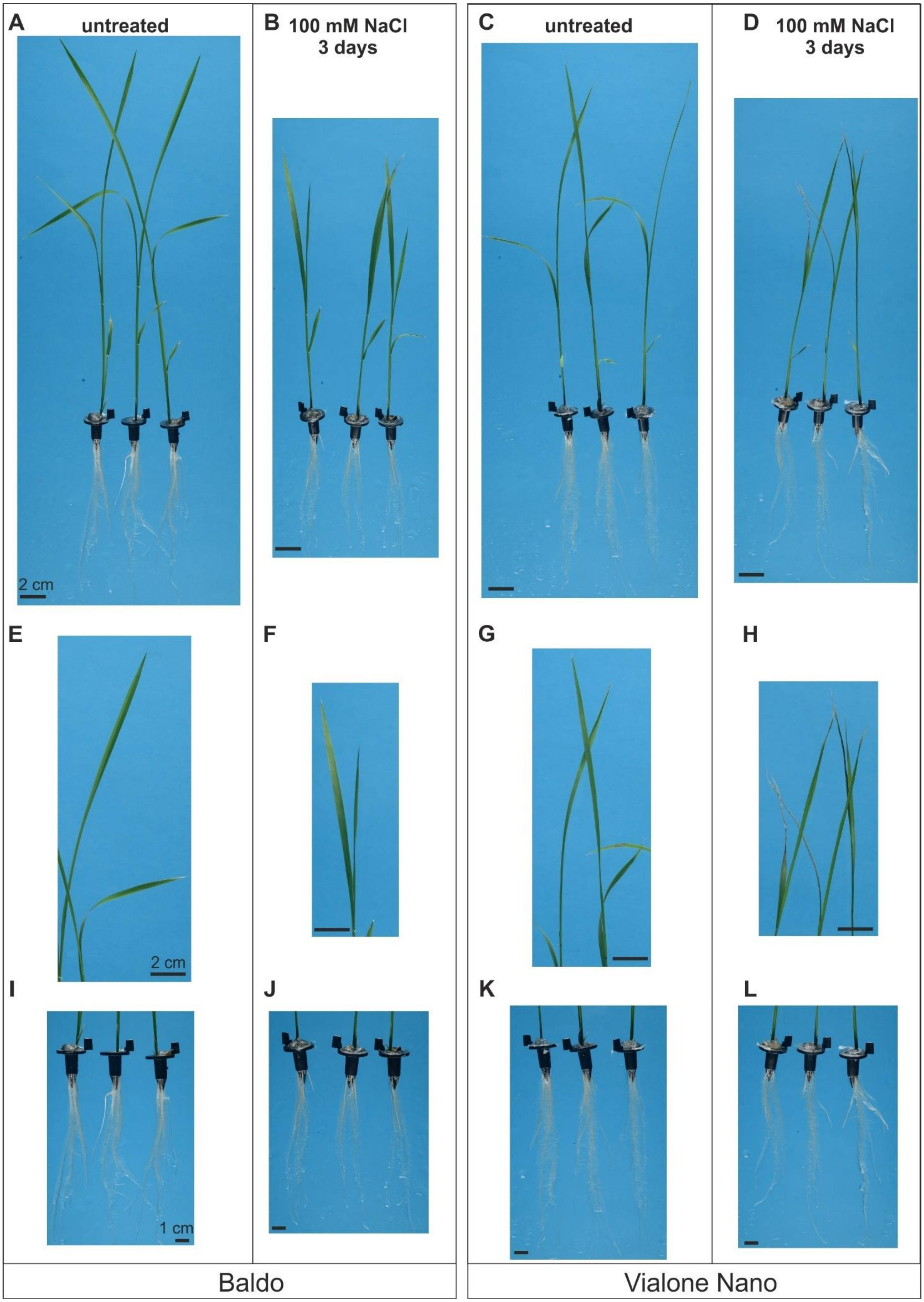
A untreated
B 100 mM NaCl 3 days
C untreated
D 100 mM NaCl 3 days
2 cm
E
F
G
H
2 cm
I
J
K
L
1 cm
Baldo
Vialone Nano

Supplementary Figure 3. Baldo and Vialone Nano untreated and treated plants after 3 days of stress. A-D) Plants are supported by seed-holders at the base. This system facilitates an easy sampling of roots and leaves. Aerial part of treated plants (B and D) are less developed than untreated plants (A and C). E-H) Second and third leaves of plants in A-D. Wilting and yellowing are visible for Vialone Nano plants (H). I-L) Roots of plants in A-D. Roots of treated plants (J and L) are shorter than control plants (I and K).

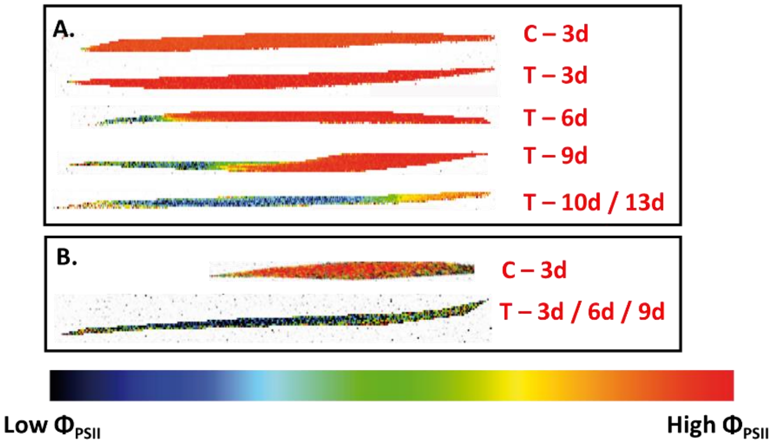

Supplementary Figure 4. Fluorescence images depicting PSII maximum quantum yield values for the whole second leaf area of Baldo (A) and Vialone Nano (B) plants. The region on the left represents the apical part of the leaf. Plants not exposed to salt treatment - C; Plants exposed to salt treatment – T. Fluorescence measurements used for the photosynthesis evaluation were performed using an imaging apparatus (see M & M) which enables monitoring the behaviour of different parts of a leaf to be monitored. The images of the PSII maximum quantum yield of plants under salt stress clearly show that the decrease in Φ_{PSII} for B plants was not homogeneous. In the sensitive variety, this differentiation was not observed, and the entire leaf showed a strong, homogeneous decrease in Φ_{PSII}.

Baldo

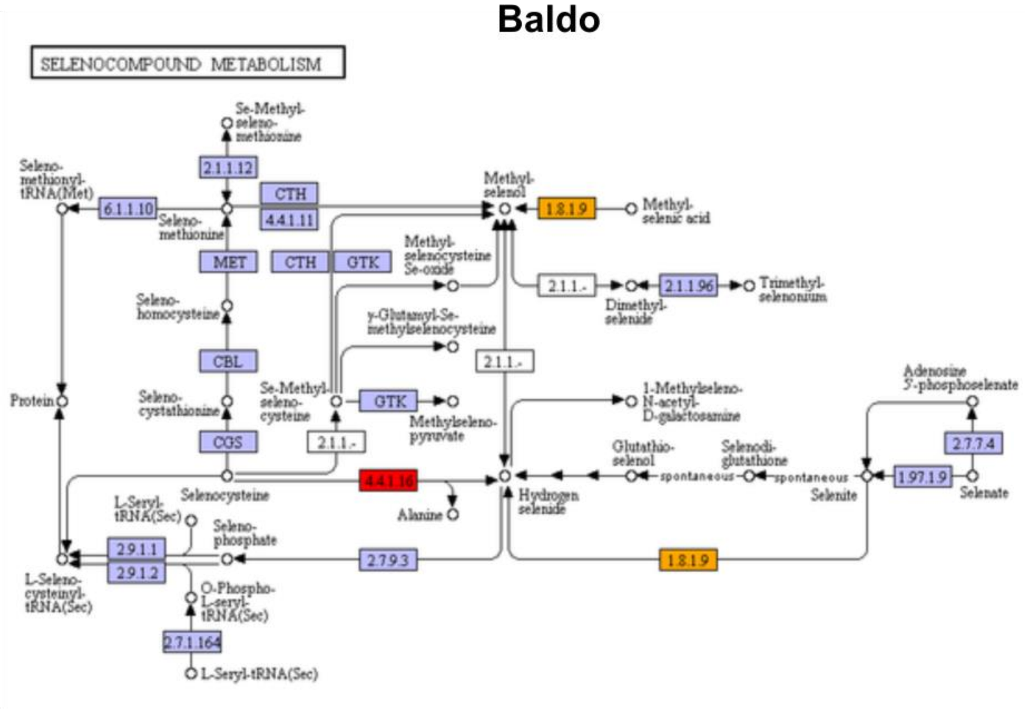

Vialone Nano

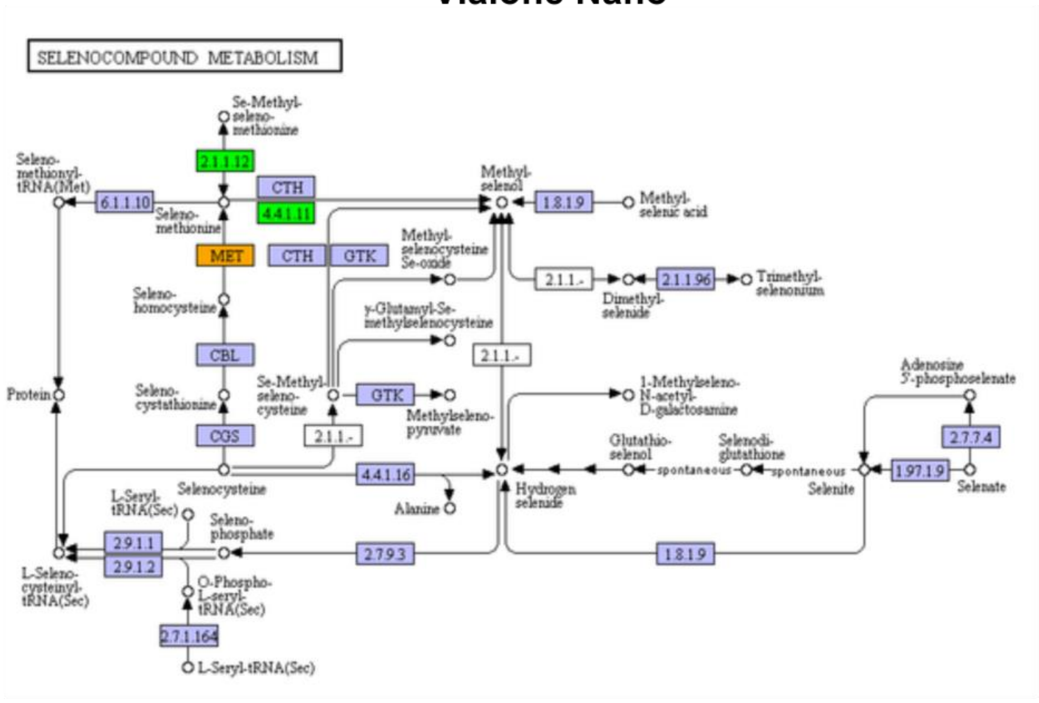

Supplementary Figure 5. Selenocompound metabolism in leaves. Colour code: green, all DEGs are up; red, all DEGs are down, Orange, DEGs are either up or down.

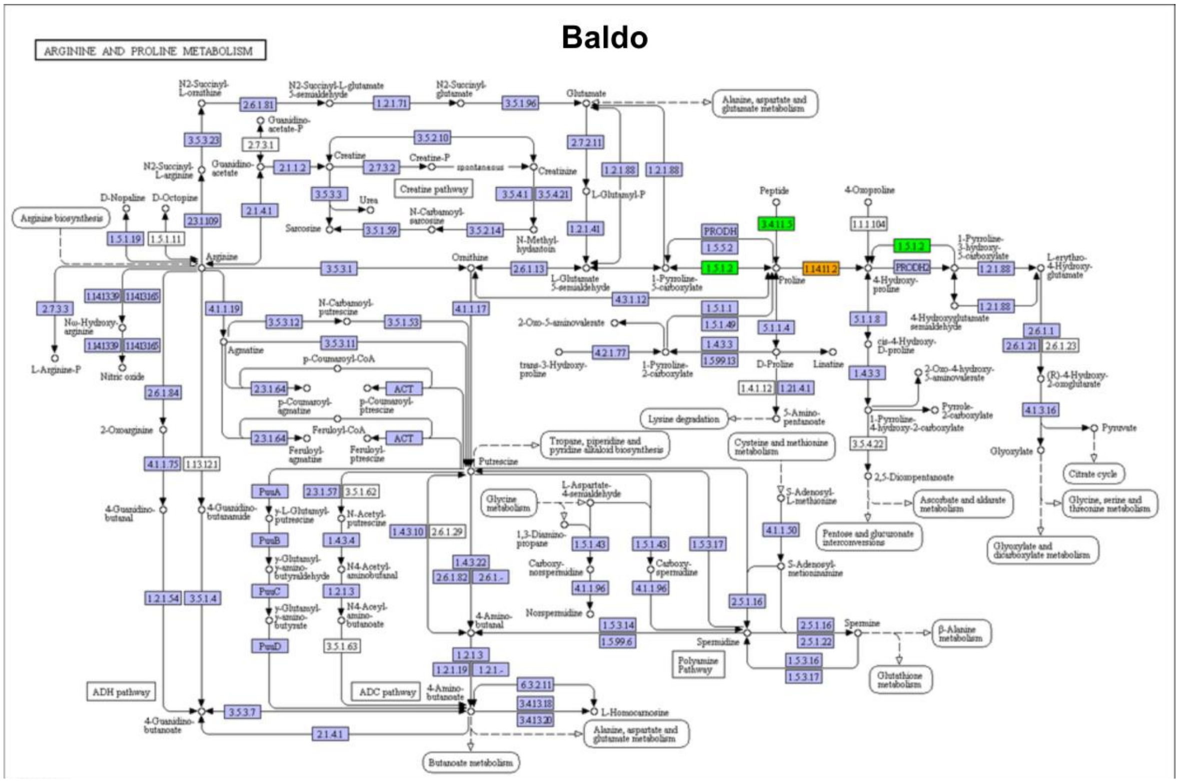

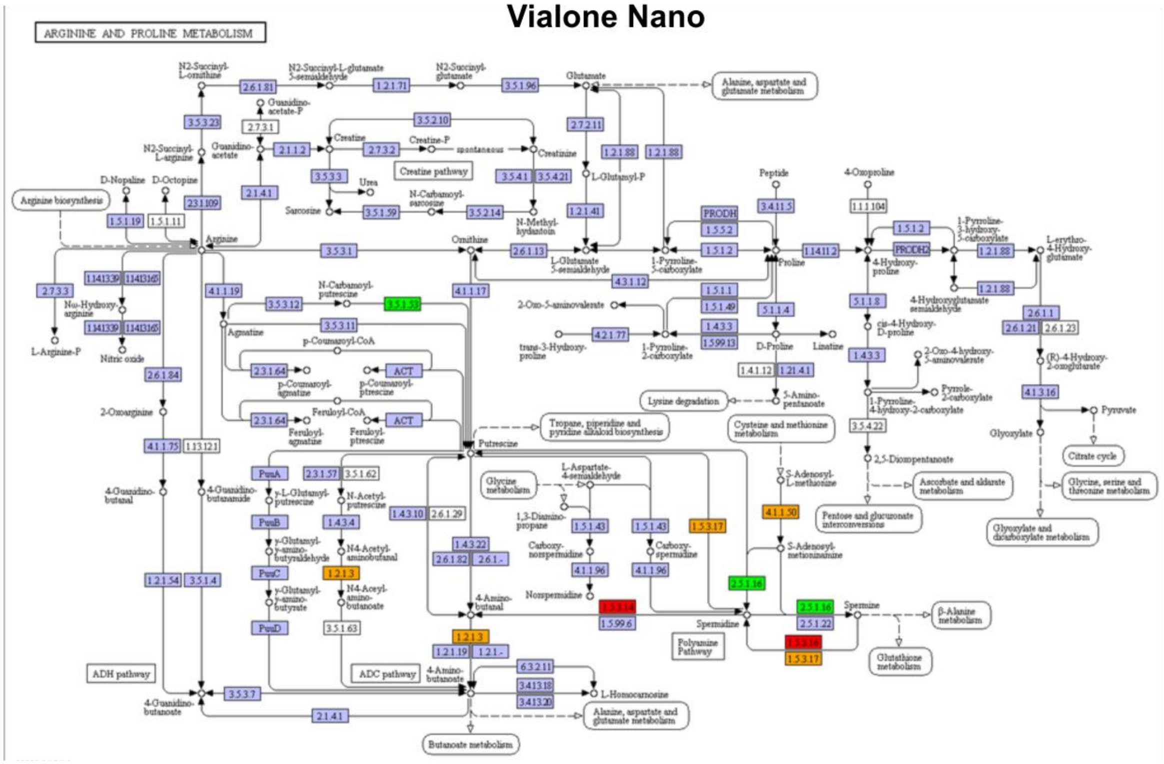

Supplementary Figure 6. Arginine and proline metabolism in leaves. Colour code: green, all DEGs are up; red, all DEGs are down, Orange, DEGs are either up or down.

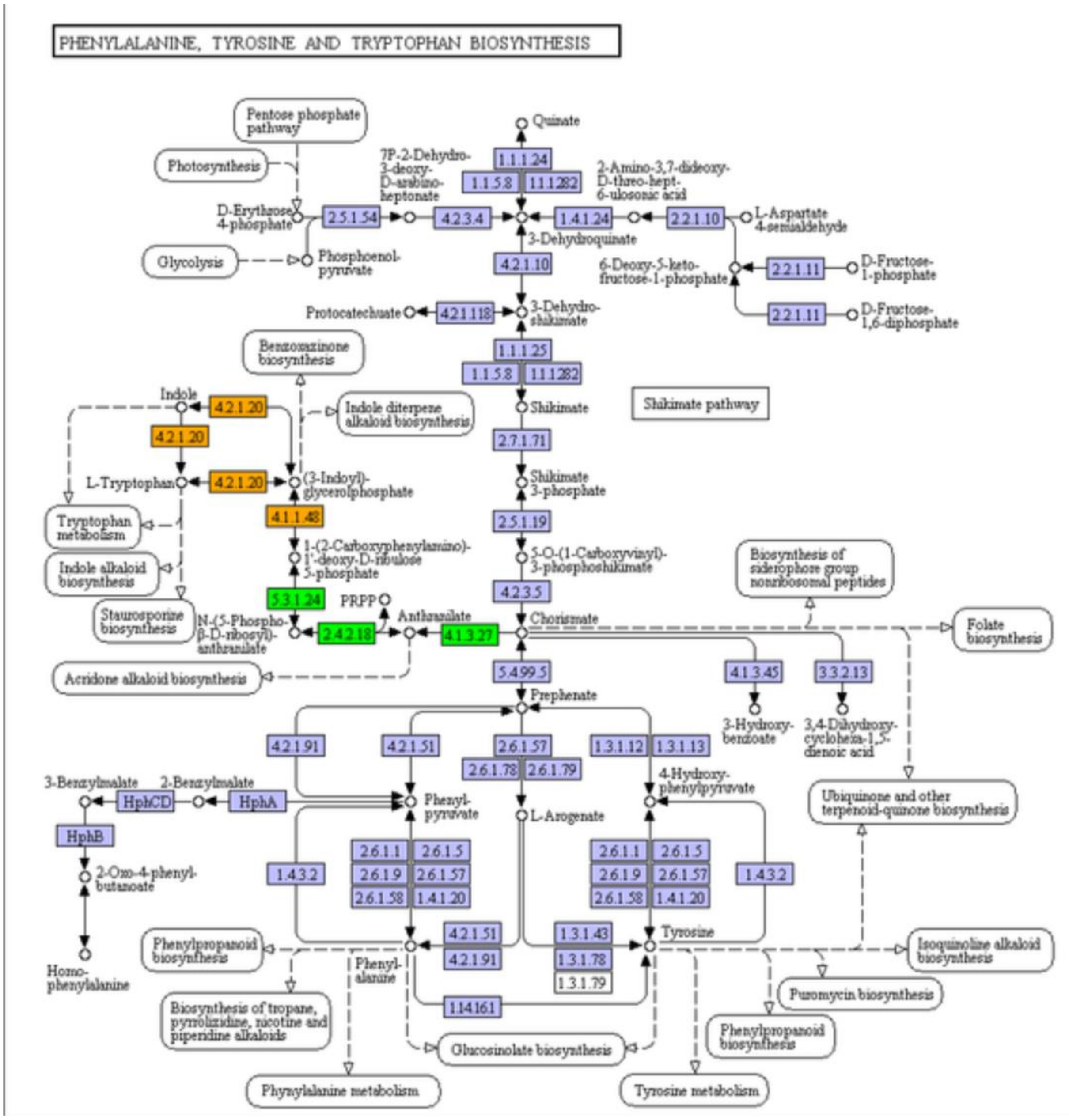

Supplementary Figure 7. Phenylalanine, Tyrosine and Tryptophan biosynthesis in B leaves. Colour code: green, all DEGs are up; red, all DEGs are down, Orange, DEGs are either up or down.

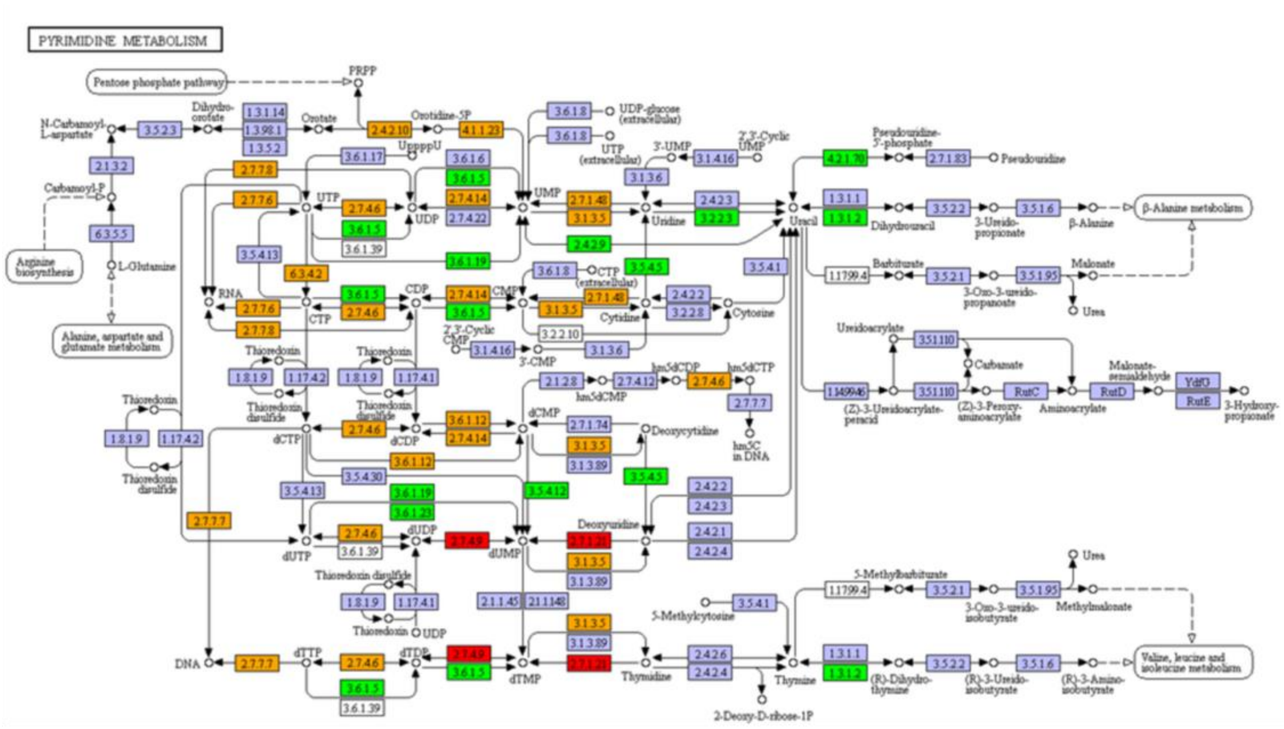

Supplementary Figure 8. Pyrimidine metabolism in B leaves. Colour code: green, all DEGs are up; red, all DEGs are down, Orange, DEGs are either up or down.

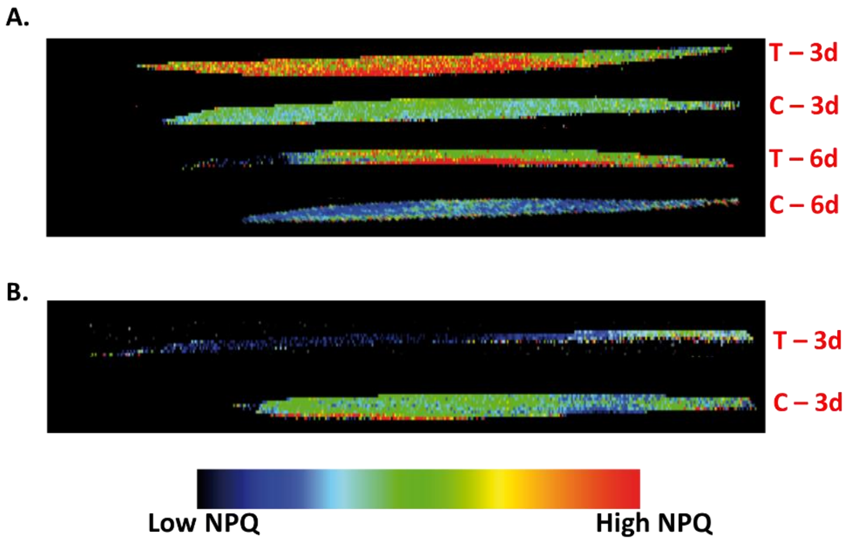

Supplementary Figure 9. Fluorescence images depicting NPQ values for the whole second leaf area for Baldo (A) and Vialone Nano (B) plants. Images were taken after 3 or 6 days of salt treatment (T), using plants not exposed to salt stress as reference (Control – C). The region on the left represents the apical part of the leaf.

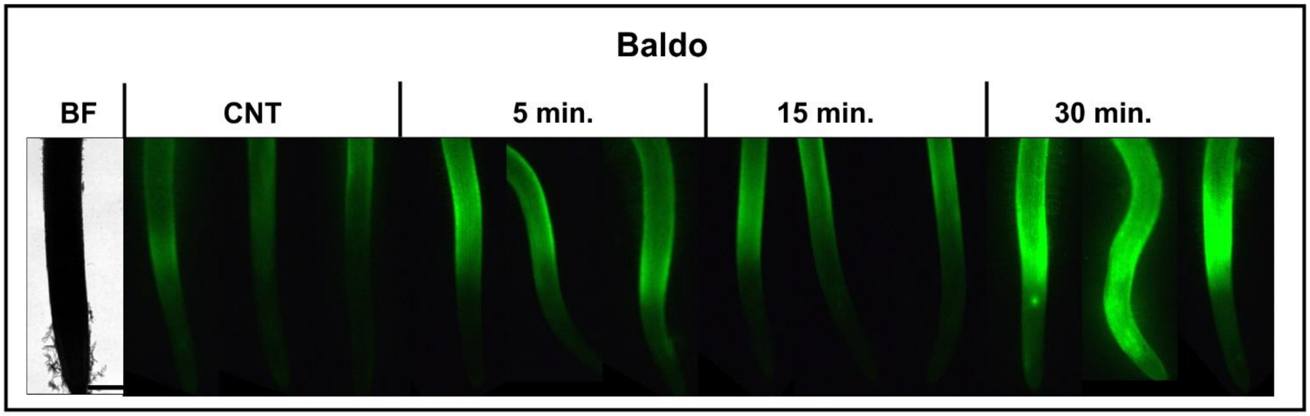

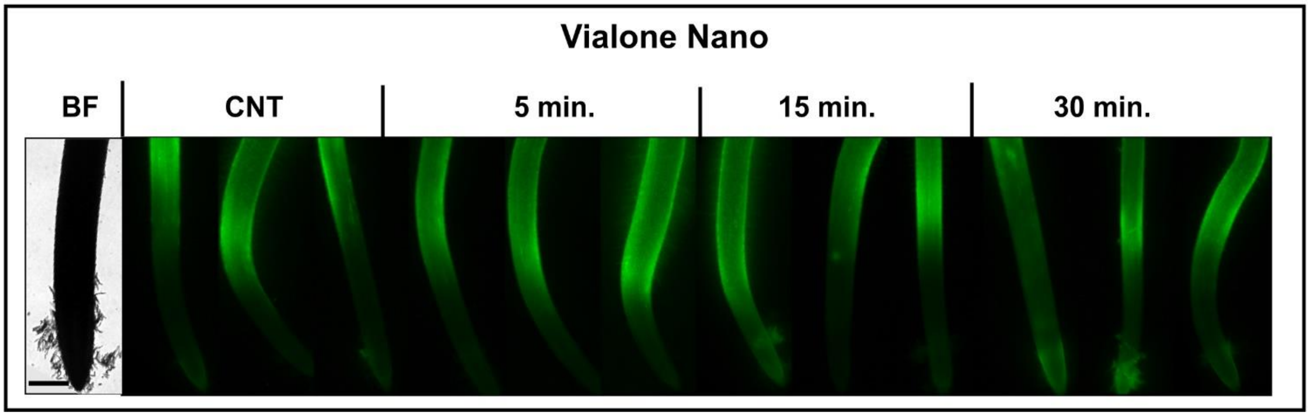

Supplementary Figure 10. Intracellular H_2O_2 measurements in 4-day old rice seedlings. Examples of pictures used for the analysis showed in Fig. 8. BF: bright field; bar = 500 μM.

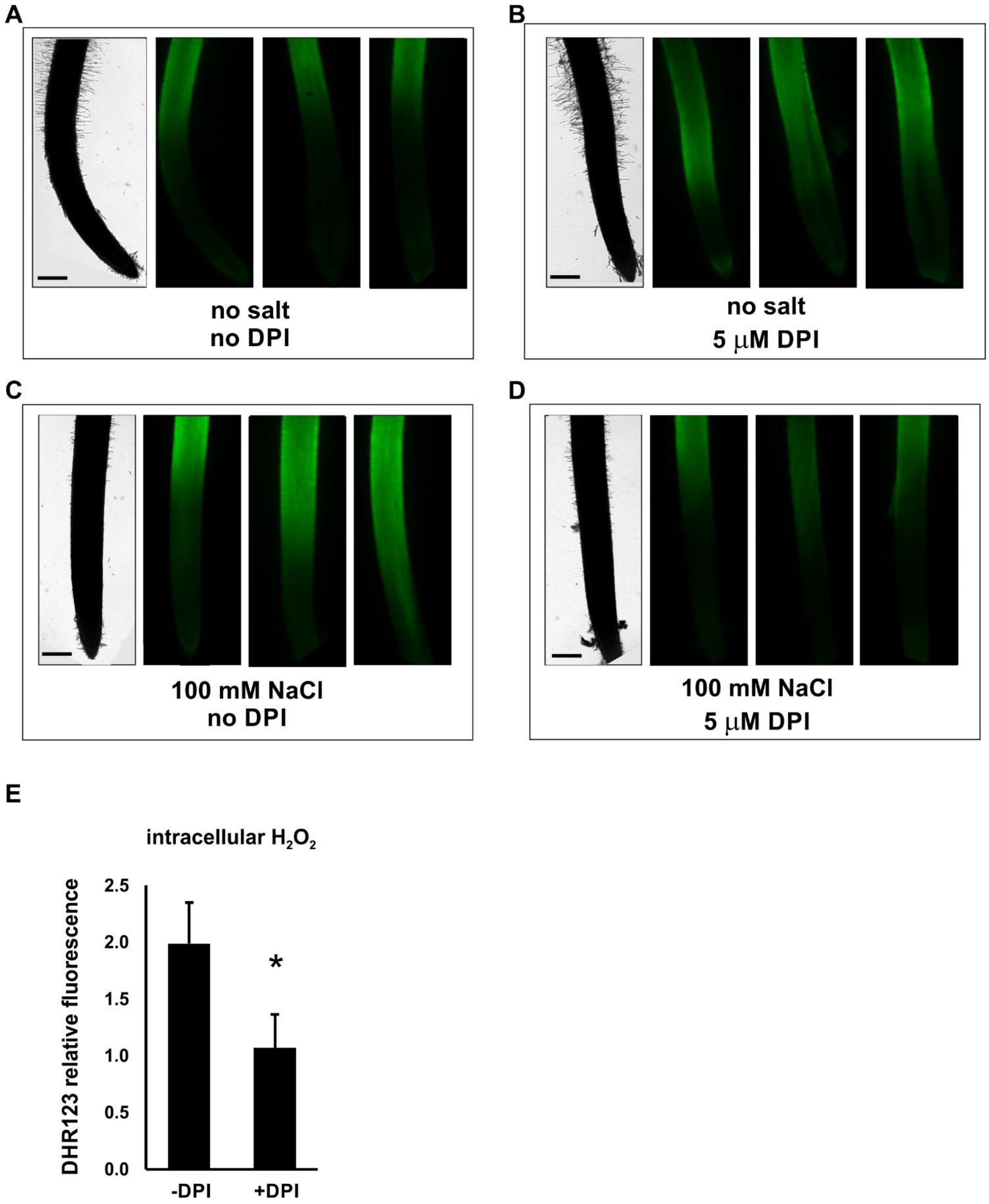
A
no salt
no DPI
B
no salt
5 μM DPI
C
100 mM NaCl
no DPI
D
100 mM NaCl
5 μM DPI
E
intracellular H2O2
DHR123 relative fluorescence
2.5
2.0
1.5
1.0
0.5
0.0
*
-DPI
+DPI

Supplementary Figure 11. Intracellular H_2O_2 measurements in Baldo 4-day old seedlings pre-treated with 5 μM DPI. A-D) DHR123 fluorescence of root tips of untreated (A-B) and salt-treated (C-D) seedlings, with (B-D) or without (A-C) DPI. Bar = 500 μM. E) Fluorescence relative to the control after 30 min of treatment. Values are mean ± SD of 10 biological replicates and 3 technical replicates (* $p < 0.01$).
